# Quality of Life after Brain Injury in Children and Adolescents (QOLIBRI-KID/ADO)—The First Disease-Specific Self-Report Questionnaire after Traumatic Brain Injury

**DOI:** 10.3390/jcm12154898

**Published:** 2023-07-26

**Authors:** Nicole Von Steinbuechel, Marina Zeldovich, Sven Greving, Laiene Olabarrieta-Landa, Ugne Krenz, Dagmar Timmermann, Inga K. Koerte, Michaela Veronika Bonfert, Steffen Berweck, Matthias Kieslich, Knut Brockmann, Maike Roediger, Michael Lendt, Michael Staebler, Silke Schmidt, Holger Muehlan, Katrin Cunitz

**Affiliations:** 1 Institute of Medical Psychology and Medical Sociology, University Medical Center Göttingen, Waldweg 37A, 37073 Göttingen, Germany; marina.zeldovich@med.uni-goettingen.de (M.Z.); sven.greving@med.uni-goettingen.de (S.G.); ugne.krenz@med.uni-goettingen.de (U.K.); dagmar.timmermann@med.uni-goettingen.de (D.T.); katrin.cunitz@med.uni-goettingen.de (K.C.); 2 Institute of Psychology, University Innsbruck, Innrain 52f, 6020 Innsbruck, Austria; 3 Departamento de Ciencias de la Salud de la Universidad Pública de Navarra, Campus de Arrosadía, 31006 Pamplona, Spain; laieneolabarrieta@gmail.com; 4 Instituto de Investigación Sanitaria de Navarra (IdiSNA), 31008 Pamplona, Spain; 5 cBRAIN, Department of Child and Adolescent Psychiatry, Psychosomatics, and Psychotherapy, LMU University Hospital, Ludwig-Maximilian University, Nussbaumstrasse 5, 80336 Munich, Germany; inga.koerte@med.uni-muenchen.de; 6 Department of Pediatric Neurology and Developmental Medicine, LMU Center for Development and Children with Medical Complexity, Dr. von Hauner Children’s Hospital, LMU University Hospital, Haydnstr. 5, 80336 Munich, Germany; michaela.bonfert@med.uni-muenchen.de; 7 Specialist Center for Paediatric Neurology, Neurorehabilitation and Epileptology, Schoen Klinik, Krankenhausstraße 20, 83569 Vogtareuth, Germany; sberweck@schoen-klinik.de; 8 Department of Paediatric Neurology, Hospital of Goethe University, Theodor-Stern-Kai 7, 60590 Frankfurt am Main, Germany; matthias.kieslich@kgu.de; 9 Interdisciplinary Pediatric Center for Children with Developmental Disabilities and Severe Chronic Disorders, Department of Pediatrics and Adolescent Medicine, University Medical Center, Robert-Koch-Str. 40, 37075 Göttingen, Germany; knut.brockmann@med.uni-goettingen.de; 10 Department of Pediatric Intensive Care Medicine and Neonatology, University Hospital Muenster, Albert-Schweitzer-Campus 1, 48149 Muenster, Germany; maike.roediger@ukmuenster.de; 11 Neuropediatrics, St. Mauritius Therapeutic Clinic, Strümper Straße 111, 40670 Meerbusch, Germany; lendt@stmtk.de; 12 Hegau-Jugendwerk GmbH, Neurological Rehabilitation Center for Children, Adolescents and Young Adults, Kapellenstr. 31, 78262 Gailingen am Hochrhein, Germany; michael.staebler@hegau-jugendwerk.de; 13 Department of Health and Prevention, University of Greifswald, Robert-Blum-Str. 13, 17487 Greifswald, Germany; silke.schmidt@uni-greifswald.de (S.S.); holger.muehlan@uni-greifswald.de (H.M.)

**Keywords:** disease-specific health-related quality of life (HRQoL), traumatic brain injury (TBI), patient-reported outcome measure (PROM), children and adolescents

## Abstract

The subjective impact of the consequences of pediatric traumatic brain injury (pTBI) on different life dimensions should be assessed multidimensionally and as sensitively as possible using a disease-specific health-related quality of life (HRQoL) instrument. The development and psychometrics of the first such self-report questionnaire for children and adolescents after TBI are reported here. Focus group interviews with children, adolescents, and their parents, cognitive debriefing, item pool generation and reduction using Delphi expert panels were performed. The resulting version was psychometrically tested on 300 individuals aged 8–17 years. After item reduction based on factor analyses, differential item functioning, reliability, and validity were investigated. The final 35 items were associated with six scales (Cognition, Self, Daily Life and Autonomy, Social Relationships, Emotions, Physical Problems). Internal consistency and construct validity were satisfactory. Health-related Quality of life (HRQoL) was significantly lower in older and in female participants, as well as those with cognitive disabilities, anxiety, depression and post-concussion symptoms, than in comparative groups. The new QOLIBRI-KID/ADO is a comprehensive, multidimensional, reliable, and valid instrument, comparable in content and items to the QOLIBRI adult version. Therefore, disease-specific HRQoL can now be measured across the lifespan and may support the amelioration of treatment, care, rehabilitation, and daily life of children and adolescents after TBI.

## 1. Introduction

Pediatric traumatic brain injury (pTBI) constitutes an important global health challenge, as it is the most common injury-related cause of death and disability in this age group [1]. The incidence and mortality rates vary widely between countries. Incidence extends from 12 (Sweden) to 670 (Germany) children per 100,000 children per year [2]. Mortality ranges from 0.5 (Germany) [3] to 3.75 (Australia) [4]. Due to medical advances, mortality has decreased over the past decades [3]. However, many surviving children and adolescents still suffer from short- and long-term functional [5], cognitive [6], emotional [7], and social impairments [6]. There has been extensive research on the manifold sequelae after pTBI using a variety of predominantly unidimensional measures and methods (e.g., [8]). The heterogeneity and complexity of TBI and the wide spectrum of affected life dimensions and negative consequences cannot be captured comprehensively using unidimensional instruments. Therefore, the perspectives of children and adolescents on their health-related quality of life (HRQoL) after TBI, as well as those of their parents (and health care professionals), should be captured in a multidimensional way [8].

The HRQoL construct is inherently multidimensional. The WHO defines it as “the individual’s perception of their position in life in the context of the culture and value systems in which they live and in relation to their goals, expectations, standards and concerns” [9]. In determining the subjective effects of health conditions on an individual’s wellbeing and functioning, a distinction is made between generic [10] and disease-specific HRQoL [11]. Generic HRQoL includes a wide range of life dimensions related to different health conditions that can be assessed with the same instrument [10]. Disease-specific HRQoL, on the other hand, concentrates on a specific health condition. Disease-specific measures are reported to be more sensitive to the consequences of a specific health condition than generic ones [12]. Until now, HRQoL in pTBI could only be measured generically using e.g., the Pediatric Quality of Life Inventory (PedsQL) [13], as there was no TBI-specific patient-reported outcome measure (PROM) for assessing HRQoL [14].

Research results on the factors associated with impaired HRQoL after pTBI have been inconclusive. Only a few studies have investigated the influence of sociodemographic variables on HRQoL after pTBI, including age at injury, (e.g., [15]), sex [16], gender [17], and education [15]. More often, associations have been reported regarding TBI severity (e.g., [15,18]) and posttraumatic stress disorder (e.g., [19]). Mental health issues such as depression (e.g., [20]) and a higher number of post-concussion symptoms (e.g., [21]) have also been linked to lower HRQoL.

For some children and adolescents after TBI, it can be challenging to obtain reliable information using self-reports. Judgements may fluctuate due to young age, including difficulties in understanding the constructs being assessed [14,22], cognitive deficits (e.g., [22]), and a possible lack of awareness [23]. Hence, proxy-reports provided by parents are often used to collect information on the HRQoL of the children in question (e.g., [24]). However, disparities between these reports are observed. Only when children cannot answer for themselves should parent ratings be collected as a surrogate [25]. Some studies [14,22] have shown that children as young as five years old are already able to report on their HRQoL, supporting the feasibility and importance of assessing the child’s perspective through self-reports.

Thus, the aim of the current study was to present the theory- and data-driven development and psychometric validation of the first TBI-specific pediatric PROM for HRQoL assessment: the Quality Of Life after Brain Injury in Children and Adolescents (QOLIBRI-KID/ADO) questionnaire—an age-adapted HRQoL instrument for children and adolescents (8–17 years) after TBI. The QOLIBRI-KID/ADO will enable the assessment of the impact of the sequelae of TBI on disease-specific HRQoL in children and adolescents, including the effects of short- and long-term treatment, interventions, and care. The contents of the selected items should be comparable to those of the adult version of the QOLIBRI, in particular to permit a longitudinal assessment from childhood to adulthood. The importance of its longitudinal clinical use is underlined by several studies reporting that some negative consequences of TBI in early childhood may not be observed until later in life (e.g., [26]). The methods used to develop the instrument will allow disease-specific HRQoL to be measured across the life span. Implementing the QOLIBRI-KID/ADO in daily clinical routine and research may support the amelioration of rehabilitation and daily life of children and adolescents after TBI.

## 2. Materials and Methods

### 2.1. Participants

The study was conducted in Germany from April 2017 to January 2022. Participants were included in this retrospective study according to the following criteria: aged 8–17 years at the time of study enrollment, diagnosis of TBI (at least three months and no more than ten years post injury), TBI severity (assessed using the Glasgow Coma Scale (GCS) at time of injury [27], International Statistical Classification of Diseases and Related Health Problems (ICD-10) [28] code, or from the clinical description of TBI severity), outpatient status (or at the start of resuming inpatient treatment), and the ability to understand and answer the questions.

Exclusion criteria were a current vegetative state, spinal cord injury, severe mental illness before TBI (e.g., psychosis, autism), epilepsy prior to TBI, disease leading to death, or very severe polytrauma (information collected by investigators from the medical records). Written informed consent was obtained from all participants and their parents. Assessments were conducted via face-to-face interviews with children and adolescents (online or in person), and parent reports were carried out in writing by mail or completed during a visit to the recruiting clinic, rehabilitation center, or outpatient center.

Sample size estimation was performed for the planned analyses, resulting in the requirement of around 140 subjects per age group (taking into account an excellent congruence coefficient of 0.98) for parameters of factor analyses with six factors according to a simulation study [29].

### 2.2. Ethical Approval

The QOLIBRI-KID/ADO study was conducted in accordance with all relevant laws in Germany, including but not limited to the ICH Harmonized Tripartite Guideline for Good Clinical Practice (ICH GCP) and the World Medical Association Declaration of Helsinki (“Ethical Principles for Medical Research Involving Human Subjects”). The study attained ethical clearance at each recruitment center, and informed consent was obtained from all participants according to the German law for data protection (General Data Protection Regulation, GDPR). The Ethics Committee of the University Medical Center Goettingen approved the study (application no. 19/4/18).

### 2.3. Sociodemographic and Clinical Data and Disability Rating

Data on participants’ age and gender as well as parents’ educational background were obtained from parent questionnaires. Information on the existence of cerebral lesions was extracted from neuroimaging data (CT or MRI) in the medical records. Severity of TBI (mild: GCS 13-15; complicated mild including detectable intracranial abnormalities in CT or MRI imaging [30]: GCS ≥ 13; moderate: GCS 9-12, severe: GCS ≤ 8) as assessed by the GCS [27] or according to the ICD-10 [28] was retrieved from the medical records. If GCS was missing, a clinical description of the TBI was aggregated from data on loss of consciousness (LOC), post-traumatic amnesia (PTA), need for ventilation and resuscitation, nausea/vomiting, post-traumatic epilepsy, presence of lesions (based on MRI/CT findings), need for surgical intervention, and injury severity score (ISS). Time since TBI was determined using information from the medical records and dichotomized using a median split to obtain two equal groups for further analyses.

Children’s and adolescents’ current health problems were reported by parents. We aggregated “physical, sensory, and cognitive problems” after TBI (smell, taste, vision, hearing, learning problems, seizures, difficulties with language/speech, back pain, difficulties moving hands/arms, walking problems, and seizures) into a summary variable. The variable “cognitive problems after TBI” comprised learning problems and difficulties with language/speech.

Functional status was rated by the investigators using the King’s Outcome Scale for Closed Head Injury (KOSCHI) [31] covering the following levels of disability/recovery after pTBI: 1 = ‘dead’, 2 = ‘vegetative state’, 3a = ‘lower severe disability’, 3b = ‘upper severe disability’, 4a = ‘lower moderate disability’, 4b = ‘upper moderate disability’, 5a = ‘good recovery’, or 5b = ‘intact recovery’.

### 2.4. Patient-Reported Outcome Measures (PROM)

The QOLIBRI-KID/ADO is a TBI-specific HRQoL self-report questionnaire for children and adolescents aged 8–17 years, containing 35 items on six scales. Satisfaction with cognitive and social functioning, self-concept, and perceived autonomy are assessed using a five-point Likert-type scale (“Not at all” to “Very”), as well as feeling bothered by emotional and physical problems. The timeframe of the evaluation is the preceding week, including the day of the assessment. Items regarding “feeling bothered” are recoded positively to allow aggregation with the satisfaction items for further analysis. Scores for each QOLIBRI scale are obtained by a linear transformation of the scores to a scale from 0 to 100 and the subsequent calculation of the mean per scale. The total score is calculated as the average scores across scales. Higher scores indicate a better HRQoL.

The generic Pediatric Quality of Life Inventory (PedsQL^TM^) [13] assesses 23 items (physical, emotional, social, and school functioning). Two total scores, a Physical Health Summary Score (8 items) and a Psychosocial Health Summary Score (15 items), can be calculated. Participants filled in the questionnaire concerning problems during the last four weeks using a four-point Likert-type scale (“Never” to “Almost always”). Scores range from 0 to 100, with higher scores indicating better HRQoL.

The Generalized Anxiety Disorder 7 (GAD-7) [32] measures seven symptoms of generalized anxiety according to the DSM-IV [33], rated on a four-point Likert-type scale ranging from 0 to 3 (“Not at all” to “Nearly every day”). The severity is characterized by minimal (1–4), mild (5–9), moderate (10–14), and severe (15–21) symptoms.

The Patient Health Questionnaire 9 (PHQ-9) [34] captures the persistence and severity of major depression with nine symptoms according to DSM-IV [33] criteria. The response categories range from 0 to 3 (“Not at all” to “Nearly every day”). Severity corresponds to the following scores: minimal (1–4), mild (5–9), moderate (10–14), and severe (15–27) symptoms.

The screening for anxiety and depression was based on parental reports, as self-reports have not been validated for children. Cut-offs for the categorization of mild, moderate, and severe symptoms were used as in other studies with parents of children aged from 5 to 12 years [35] and adolescents [36].

The German translation of the Post-Concussion Symptom Inventory (PCSI-SR8 for children and PCSI-SR13 for adolescents) [37] includes 16 self-rated post-concussion symptoms for children and 21 for adolescents. The PCSI-SR8 and PCSI-SR13 are answered on a Gutmann-type scale ranging from 0 to 2 (“No” to “A lot”) and from 0 to 6 with three anchor categories (“Not a problem”, “Moderate problem”, and “Severe problem”), respectively. Total scores are computed as the sum of all PCSI scales (physical, emotional, cognitive, and sleep/fatigue) for the post-TBI evaluations and categorized as being above, below, or within the age group’s average range (*M* ± 1*SD*).

### 2.5. Neuropsychological Test

The Rey Auditory Verbal Learning Test (RAVLT) [38] tests the ability to acquire 15 words in eight trials. It includes five consecutive recall trials (Trials I–V). The learning rate is obtained by subtracting the number of words correctly recalled in Trial I from those in Trial V. Learning rates are compared with the respective age norms [38] and categorized as being above, below, or within the average range (*M* ± 1*SD*).

### 2.6. Psychometric Item Reduction, Differential Item Functioning, Reliability and Validity Analyses, and Descriptive Statistics of the QOLIBRI-KID/ADO Questionnaire

In reporting on questionnaire development and validation, we followed the Consensus-based Standards for the Selection of Health Measurement Instrument (COSMIN) guidelines and checklists [39]. The original item pool, consisting of 83 items for children and 87 items for adolescents, was based on the results of focus group interviews, items adapted from (HR)QoL and TBI-related instruments, and a Delphi panel consensus procedure. Forty-three items that were conceptually and semantically acceptable and mostly comparable to the content of the adult version of the QOLIBRI were retained. The detailed procedure is described in the Supplementary File S1.

The number of items was then reduced by carrying out the following psychometric analyses. First, confirmatory factor analysis (CFA) was performed assuming the scale structure of the QOLIBRI adult version, which was supported by the results of the focus group interviews with children and adolescents after TBI [14]. Diagonally weighted least squares with mean and variance adjustment (WLSMV) was performed as an estimation approach suitable for ordinal data [40]. The following indices were applied for model evaluation (cut-off values in parentheses): χ^2^ statistics with respective *p*-values (*p* > 0.01) [41], comparative fit index (CFI > 0.95) [42], Tucker–Lewis index (TLI > 0.95) [42], root mean square error of approximation (RMSEA < 0.06) [43] with a 90% confidence interval (CI90%), and standardized root mean square residual (SRMR < 0.08) [42]. Item loadings on the assigned latent factors above 0.40 were considered satisfactory [44]. Items were then analyzed in terms of their contribution to the internal consistency of the assumed home scale as measured by Cronbach’s α when the item was omitted. Items that did not increase the initial internal consistency of the home scale after omission were retained. A Cronbach’s α ≥ 0.70 was regarded as being satisfactory [45].

Since two age-adapted versions were administered, it was investigated whether the response behavior of the participants warranted an aggregation of the two versions. A logistic ordinal regression approach was applied to detect differential item functioning (DIF) (LORDIF) [46]. The DIF evaluation consisted of a comparison of a LORDIF model that included scale scores for the item alone and a LORDIF model that included the scale score, the age category, and the age category–ability interaction. Items exhibit a DIF when a significant difference (α = 0.01) is identified between the LORDIF models and when the associated effect size (McFadden’s pseudo R^2^) indicates more than a very small effect (i.e., r > 0.05) [47]. In the absence of DIF, responses were treated as being independent of age and further analyses could then be performed using an aggregated age sample.

After reducing the number of items using the CFA and obtaining evidence for the absence of DIF between age versions, means (*M*), standard deviations (*SD*), and skewnesses (*SK*) were calculated for the individual items, as well as the total and the scale scores. Skewness was considered symmetrical for values from −0.5 to 0.5, moderately skewed for values from ±0.5 to ±1, and heavily skewed for values beyond ±1 [48]. Furthermore, the percentage of responses in the most satisfied/least bothered category (ceiling effects) was reported, as well as the percentage of responses in the least satisfied/most bothered category (floor effects).

The internal consistency of the aggregated final item set was again investigated using Cronbach’s α and corrected item-total correlations (CITC). As measuring HRQoL in subjects with cognitive impairment may pose a threat to internal consistency [49], Cronbach’s α was also assessed separately in individuals with and without cognitive problems. To further investigate the reliability of the questionnaire across different domains of symptoms, Cronbach’s α was calculated and stratified by learning rate as measured by the RAVLT (low: *M −* 1*SD* vs. average-to-high: *M* and *M +* 1*SD*), TBI severity (mild vs. moderate/severe), functional recovery (KOSCHI, low: <5 vs. full score: 5), presence of physical, sensory, and cognitive problems (yes vs. no), post-concussion-symptoms (PCSI-SR8/-SR13, higher symptom frequency: *M* + 1*SD* vs. average to low symptom burden: *M* and *M* − 1*SD*), and different levels of depression and anxiety (PHQ-9 or GAD-7, mild to severe: ≥5 vs. no symptoms: <5). Values of Cronbach’s α ≥ 0.60 were considered satisfactory [45] and items yielding a CITC > 0.40 were kept [50].

Test-retest reliability was examined in a subsample of 28 participants, who filled in the instrument 10 to 20 days after the initial self-report. Intraclass correlation coefficients were computed for the total score and each scale based on a two-way random effects model without aggregation (ICC-2,1). An ICC above 0.60 indicated satisfactory reliability [51]. Furthermore, the standard error of measurement (SEm) [52] and the variation between test and retest were calculated, as well as the minimal/smallest detectable change (MDC/SDC), which indicates the questionnaires’ ability to capture differences across time. In line with what is generally known about the stability of HRQoL assessments over time [53], changes below 15% of the total possible range of a measurement instrument were regarded as acceptable [54]. On the five-point response scale used in the QOLIBRI-KID/ADO, the cut-off would thus be MDC ≤ 0.75.

Then, a CFA was again performed using the final item set to determine the factorial structure of the instrument and the model fit to the data. Fit indices (i.e., χ^2^ statistics, CFI, TLI, RMSEA, and SRMR) were inspected, and the assumed one-level six-factor model of the QOLIBRI adult version [55,56] was compared with a two-level six-factor model with an additional latent global HRQoL factor by means of a chi-square test.

To investigate whether the QOLIBRI-KID/ADO reflects the construct of HRQoL, we calculated Pearson correlation coefficients for total and scale scores with the generic PedsQL, as there is no other pTBI-specific HRQoL questionnaire. In addition to the conventional scale scores, a psychosocial functioning score for the QOLIBRI was aggregated by averaging the cognitive, self, social, and emotional scale scores and correlated with the PedsQL psychosocial functioning score. Cohen’s cut-off criteria for evaluating the strength of associations were applied: small (0.10), moderate (0.30), and large (0.50) [57]. The correlations (*r* ≥ 0.30) between the QOLIBRI-KID/ADO total and scale scores and the PedsQL scores were expected to be at least moderate.

The QOLIBRI-KID/ADO total and scale scores were correlated with the GAD-7 and the PHQ-9, expecting small to medium Pearson correlations [57]. Children and adolescents with no or minimal anxiety and depression (scores 0–4) were compared with those reporting at least mild anxiety or depression (scores ≥ 5). Higher symptom burden was assumed to be associated with lower HRQoL, as found in children and adolescents with multiple chronic diseases and mental health issues [58].

Finally, the association between sociodemographic variables (i.e., age, gender, education of the parents) and the QOLIBRI-KID/ADO total and scale scores was investigated. Based on earlier pediatric studies, we assumed that boys would report higher HRQoL values than girls [17] and that HRQoL would decrease with increasing age [59]. The following clinical characteristics were assumed to be associated with lower HRQoL, as in the respective reference group: moderate to severe TBI [18], lower level of recovery [5], high physical, sensory, and cognitive problems [59], and high post-concussion burden [21]. To investigate symptom burden descriptively, the frequency of symptoms grouped by domain was first examined for the total sample. Then, the different symptom domains grouped by TBI severity (mild vs. moderate/severe) were analyzed to better characterize the population on which the psychometric development of the questionnaire was based. Cronbach’s α analyses were conducted here as well. The same cut-off values were applied as used for the total and scale scores of the total samples.

Pearson correlation analysis was then performed, investigating the relationship between time since TBI (in months) and the QOLIBRI-KID/ADO total score. Also, the presence of persisting neuropsychiatric symptoms (sequelae) and the mean time since TBI were compared between participants with and without neuropsychiatric symptoms. Any parent-reported cognitive problems, mild to severe depression or anxiety, and above-average post-concussion symptoms were regarded as neuropsychiatric symptoms.

Pearson’s correlation coefficient was used in the correlational analyses, Student’s *t*-test for mean comparisons, and Cohen’s *d* for effect size calculations in mean difference analyses. The association between the QOLIBRI-KID/ADO total score and age was analyzed using significance testing against the assumption of no correlation. For comparisons based on other demographic and clinical data using *t*-tests, a one-tailed significance test was applied. Cohen’s *d* values of 0.20, 0.50, and 0.80 represented small, medium, and large effects, respectively.

### 2.7. Missing Values

Little’s test of complete randomness [60] on the QOLIBRI-KID/ADO data indicated no evidence of a deviation from complete randomness (all: χ^2^(820) = 691.9, *p* = 0.999; children: χ^2^(270) = 258.44, *p* = 0.683; adolescents: χ^2^(652) = 591.51, *p* = 0.956). As no more than 5% of the data were missing, the number and distribution of missing values were considered acceptable. QOLIBRI-KID/ADO scores for individual participants were computed if not more than one-third of the scales’ items were missing, and total scores were computed only when scores were present for all scales. For the PedsQL, scale scores were only computed if up to a half of a scale’s responses were missing [61]. For the GAD-7 and PHQ-9, total scores were calculated if up to one-third of the items per scale were missing.

All analyses were performed with R (version 4.1.0) [62], using the packages naniar [63] for the analysis of missing values, lavaan [64] for CFAs, lordif [65] for DIF analyses, and psych [66] for the calculation of psychometric properties. Unless otherwise stated, the significance level was set at 5%.

## 3. Results

### 3.1. Participants

Data were collected from 302 participants (152 children and 150 adolescents), whereas data from two adolescents had to be excluded due to the violation of inclusion criteria. Most participants were male (total: 60%; KID: 62% and ADO: 57%). The mean age was 12.48 ± 2.71 years (KID: 10.63 ± 1.40; ADO: 15.24 ± 1.47). Most participants were interviewed in person (74%), the others online. Participants who were assessed online did not report a different QOLIBRI-KID/ADO total score (*M* = 75.10) than those assessed in person (*M* = 73.81; *t*(124.16) = −0.81, *p* = 0.418, *d* = 0.11). They most commonly experienced an uncomplicated mild TBI. Thirty-three percent underwent neuroimaging (CT/MRI), which identified 29% participants with at least one cerebral lesion. Nearly half of the TBIs had occurred up to four years prior to the participation in this study. Descriptive data are shown in Table 1 (see Appendix A, Table A1, for information on age groups).

As shown in Table 1, approximately one third of participants were experiencing symptoms at the time of the assessment: any physical, sensory, or cognitive problem (33%), cognitive problems only (26%), low learning rate (41%), anxiety (28%), depression (35%), and post-concussion symptoms (11%).

Table 2 differentiates between the symptoms grouped by TBI severity. Almost all symptoms were more common in individuals after moderate-to-severe TBI (24% to 54%) than in those after mild TBI (5% to 38%). Only post-concussive symptoms were distributed almost evenly between the two severity groups.

### 3.2. Psychometric Item Reduction, Differential Item Functioning, Reliability and Validity Analyses, and Descriptive Statistics of the QOLIBRI-KID/ADO Questionnaire and the Other PROMs

A total of 43 items from the child and adolescent versions were included in the psychometric item reduction analyses. Thirty of them corresponded in content to the questions from the adult QOLIBRI version (see Table 3). Cronbach’s α for the scales ranged from 0.70 to 0.78. One item was excluded due to low factor loading (CFA), and two items because the Cronbach’s α for the scales increased when they were omitted. In addition, five items, which resulted in the smallest decrease in their scale’s internal consistency when omitted, were removed to further reduce the length of the questionnaire.

Following these reductions, the final QOLIBRI-KID/ADO contained 35 items. CITCs and item loadings ranged from 0.29 to 0.77 and 0.37 to 0.81, respectively. Although CITCs of the items “Orientation”, “Daily Independence”, “Family Relationship”, and “TBI Effects” were slightly below the cut-off value of 0.40, they were kept to maintain comparability with the adult version.

Analyses of DIF showed no relevant differences between the two age groups (see Table A2 in Appendix B). Consequently, the total sample was used in the further analyses. Table 4 presents relevant questionnaire scores. For the QOLIBRI-KID/ADO, mean scores for “satisfaction” scales were higher than 50, as often observed in other satisfaction studies [67]. These scales were also moderately to highly skewed to the left. The “bothered” scales, however, yielded a mean close to 50 and no skewness. The total score was evenly distributed. Descriptive statistics of the scales by age group and the distribution of participants’ responses to the reduced item set can be found in Table A6 and Table A3, Table A4 and Table A5 in Appendix C, respectively.

Table 5 shows Cronbach’s α for the total and scale scores of the total sample and for impaired and unimpaired subgroups. All QOLIBRI-KID/ADO scores displayed good internal consistency, with Cronbach’s α ranging from 0.70 to 0.89. Reliability coefficients stratified by symptom domain were mostly satisfactory, with Cronbach’s α ≥ 0.60. For the subgroup with higher post-concussion symptoms, the internal consistency was rather poor for the “Physical Problems” and “Emotions” scales (Cronbach’s α = 0.49). However, these groups were somewhat small for a reliable alpha calculation.

Almost no items demonstrated an increase in Cronbach’s α for the home scale if an item was dropped, except for “Family Relationship”. Similarly, almost all items exhibited an acceptable CITC, except for the items “Orientation”, “Everyday Independence”, and “Family Relationship” (see Appendix D, Table A7).

For the test-retest correlations, ICCs for twenty-eight self-reports were analysed at two consecutive time points with an interval of 10 to 20 days (*M* = 11.63, *SD* = 2.84) for the scales ranging from 0.42 to 0.64, mostly slightly below to the criterion of 0.60. Table 6 shows the ICC, SEm, and MDC. The MDCs ranged from 1.53 to 2.58 indicating that all the values exceeded the 0.75 criterion.

**Figure A1:**
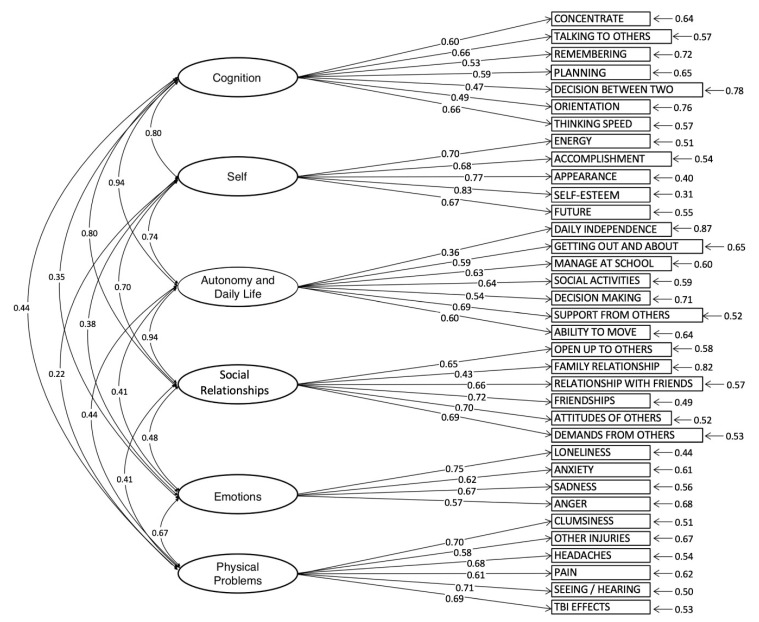
CFA model of the structure of QOLIBRI items, scales (latent factors), and path coefficients.

Inspecting the response categories revealed that the lowest response category “not at all” was not endorsed by participants for the items “Orientation” and “Accomplishment”. Therefore, prior to CFA, the response categories “not at all” and “slightly” were collapsed into one category for the satisfaction items. For the “bothered by” items, the categories “very” and “quite” were collapsed. The one-level model comprising six correlated latent factors yielded a better fit compared to the two-level model with six latent factors and an additional latent global HRQoL factor (Δχ²(9) = 125.09, *p* < 0.001). It also exhibited fit indices that met all the requirements for an excellent fit (CFI = 0.97, TLI = 0.97, RMSEA = 0.05 CI90% [0.04–0.05], SRMR = 0.08). Only the χ²-fit index displayed a significant discrepancy between the model and the data. This can, however, be attributed to the high number of degrees of freedom (df = 545) and is therefore not indicative of a model misfit. For more details on fit indices and resulting factor loadings, see Table A8 (Appendix E) and Figure A1.

The Pearson correlation coefficients for the QOLIBRI-KID/ADO total and scale scores and the comparable PedsQL scale and total scores ranged from *r* = 0.47 (psychosocial functioning scores) to *r* = 0.67 (total scores). Overall, the correlations suggested some expected overlap between the assessed generic and TBI-specific HRQoL constructs. The correlations between the scales of the QOLIBRI-KID/ADO total score and emotional states using the GAD-7 and PHQ-9 were *r* = −0.31 and *r* = −0.36, respectively. See Table 7 for further details.

Table 8 shows comparisons of QOLIBRI-KID/ADO total scores with regard to different sociodemographic and clinical groups.

Individuals without anxiety symptoms reported significantly higher QOLIBRI-KID/ADO total scores than those with mild to severe anxiety. Similarly, individuals without depression experienced significantly higher QOLIBRI-KID/ADO total scores than those with mild to severe depression.

Age was found to be negatively correlated with the QOLIBRI-KID/ADO total score, with children reporting higher QOLIBRI-KID/ADO total scores than adolescents (*r*(296) = −0.22, *p* < 0.001). Significantly higher QOLIBRI-KID/ADO total scores were seen in male than in female participants.

Comparisons of participants after mild TBI and participants after moderate to severe TBI yielded no significant differences in terms of the QOLIBRI-KID/ADO total score (see Appendix F, Table A9). However, time since TBI was weakly but positively correlated with the QOLIBRI-KID/ADO total score (*r* = 0.14, *p* = 0.014). Participants having experienced a TBI within the last four years reported significantly lower QOLIBRI-KID/ADO scores then those after a TBI dating back more than four years.

Participants with KOSCHI scores of 5a or 5b (i.e., those with good recovery) rated their total HRQoL significantly higher than did those with KOSCHI scores of 3a to 4b. In line with these findings, participants for whom parents described no problems after TBI reported a higher total HRQoL score than did children and adolescents for whom parents reported cognitive, sensory, or physical problems after TBI. Participants who experienced more post-concussion symptoms (PCSI) reported a significantly lower total HRQoL than did those with average and fewer symptoms.

In our study, at least one neuropsychiatric symptom was reported for 156 participants (see Appendix G, Table A10). Participants with cognitive problems and mild to severe anxiety did not differ in their time since TBI from those without these symptoms (see Appendix G, Table A10). Furthermore, participants who reported mild to severe depression and above-average post-concussion symptoms were those who had experienced the TBI more recently (PHQ: Δ*M* = 0.71 years, *t*(215.38) = 2.06, *p* = 0.040; PCSI: Δ*M* = 1.14 years, *t*(44.02) = 2.35, *p* = 0.023). This pattern of results suggests that our study participants reported cognitive problems and anxiety symptoms with equal frequency across the time span since TBI, whereas depression and post-concussion symptoms often seemed to occur sooner after TBI.

## 4. Discussion

The aim of the current study was to report on the development and psychometrics of a new PROM, the QOLIBRI-KID/ADO questionnaire, for assessing TBI-specific HRQoL in children (8–12 years) and adolescents (13–17 years). The longitudinal comparability of HRQoL measurement in children and adolescents was an important goal of the study. Item selection was based on satisfactory psychometric characteristics and the notion that a sufficient number of items from the adult QOLIBRI version, partly adapted for the pediatric version, could be retained to allow evaluation over the life span. Furthermore, DIF analyses did not reveal important differences between the response behaviors of children and adolescents. Thus, the two age-adapted modules were aggregated into one shortened QOLIBRI-KID/ADO version. This collated version represents an instrument that is economical (in terms of time and patient burden) and has good to excellent psychometric properties.

In this study, HRQoL is operationalized as the self-reported experience of children and adolescents’ own subjective health status, functioning, and well-being after TBI. TBI-specific aspects of HRQoL are most often not captured as sensitively by existing generic PROMs [12], and there is as yet no disease-specific, self-reported, age-adapted HRQoL instrument for children and adolescents after TBI. The QOLIBRI-KID/ADO questionnaire meets the need for a theory-driven, multidimensional instrument that is age-appropriate, reliable, and valid for children and adolescents after TBI. It is TBI-specific and addresses the unmet need to measure self-reported pediatric HRQoL after TBI. It captures multiple aspects of HRQoL, including the physical, psychological (e.g., emotional and cognitive), social, and functional domains, in a similar way to the adult version [55,56]. It therefore allows TBI-specific HRQoL to be assessed from childhood to old age using age-appropriate versions of a single instrument, a rare achievement in longitudinal observational research and clinical studies. The content of the QOLIBRI-KID/ADO questionnaire is specifically relevant for children and adolescents who have experienced a TBI, as reported in a study on focus group analyses [14].

This study reflects the good psychometric and validity properties of the new TBI-specific pediatric HRQoL questionnaire, the QOLIBRI-KID/ADO. Most of the children and adolescents were quite satisfied with their HRQoL and did not report feeling greatly bothered by physical or emotional problems. This may be partly explained by the sample composition, which included rather few individuals who experienced moderate and severe TBI [18], disability or chronic health conditions [68], cognitive impairment [15,69], or risk factors such as family violence [70]. Nevertheless, in our sample, 26% to 41% of the participants presented with symptoms in different domains at the time of the assessment: physical, sensory, or cognitive problems, a lower learning rate, anxiety, or depression as well as post-concussion symptoms (11%). Interestingly, symptoms stratified by TBI severity were distributed across all severity groups and tended to be higher following moderate to severe TBI. However, even in the group after mild TBI, a quarter to more than a third suffered from symptoms in different domains. Due to the small sample sizes in some groups, the results were not analyzed further in this study and should not be generalized. This characterization should support readers in better understanding the population in which the QOLIBRI-KID/ADO questionnaire was developed and validated.

Low socio-economic status is often associated with lower HRQoL (e.g., [59]). This pattern was also present in our study. Participants whose parents hold a university degree rated their HRQoL higher than others. However, also this finding should be treated with caution, as individuals living in households with a lower level of education were underrepresented in our study.

Despite variations in the demographic and clinical characteristics, the internal consistency remained satisfactory across all groups. The reliabilities of the QOLIBRI-KID/ADO total and scale scores are at least acceptable (Cronbach’s α ≥ 0.70), including those for subgroups with any symptom burden. Only the scales “Cognition” and “Daily Life and Autonomy” (Cronbach’s α 0.57–0.68) exhibited a somewhat lower reliability for the subgroups with actual problems (low functional recovery, physical, sensory, and cognitive problems, cognitive problems only, depression, and post-concussion symptoms), where some variance may be expected [71]. In individuals with higher rates of post-concussion symptoms, the internal consistency was below the cut-off for “Emotions” and “Physical Problems” scales. Given the small sample sizes (*N* = 34), these results should be treated with caution. This finding is compatible with several frequently used pediatric instruments which are also characterized by lower internal consistency scores (i.e., <0.60) [72]. Given the satisfactory reliability results for the total sample, the different symptom domains, and the TBI severity groups, further validation of the QOLIBRI-KID/ADO in more severely impaired individuals and those still reporting symptoms years after the TBI may shed more light on this issue. Some authors have already discussed the relationship between self-awareness and HRQoL in adult patients (e.g., [73]). However, there is a paucity of literature investigating this topic in pTBI. Nevertheless, we can conclude that our results demonstrate that the QOLIBRI-KID/ADO is sensitive to cognitive impairment and that cognitive impairment does not affect the reliability of self-reports.

The test-retest reliability was examined 10 to 20 days after initial testing, in line with recommendations for health status measures [74]. The test-retest reliability of the scales and the total score is slightly below the criterion (ICC < 0.60), but comparable to that of other pediatric instruments [51] and thus acceptable in the pediatric field.

The CFA results support this six-factor structure of the QOLIBRI-KID/ADO version, which is in line with previous studies on generic HRQoL in children [10]. This finding is congruent with multiple analyses of the adult version of the QOLIBRI, which indicate a one-level model with six latent factors [75,76]. The fit indices should however be interpreted with caution, since these were established using maximum likelihood estimation techniques [77]. Therefore, an external validation of the factorial structure of the QOLIBRI-KID/ADO is recommended using additional samples.

Validity analyses revealed that the QOLIBRI-KID/ADO was positively related to a measure of generic HRQoL (i.e., the PedsQL), indicating a certain expected construct congruence [13]. It showed moderate associations with the total and the physical scales, but also, though weaker, associations with the psychosocial domains of the PedsQL.

Furthermore, we found a negative relationship between HRQoL, anxiety and depression which are common sequelae of TBI lasting for up to 10 to 20 years after pTBI in adults [78]. Previous findings report that 11 to 45% of children are at risk of developing mental health problems after TBI (e.g., [79]). In our study, 28% of participants showed mild to severe symptoms of anxiety, and 35% mild to severe symptoms of depression, even up to 10 years after TBI, and reported a significantly reduced HRQoL. Similar results were described for children and adolescents with chronic diseases and mental health problems [58]. Early screening and therapy of emotional problems in children and adolescents after TBI could facilitate and ameliorate the rehabilitation and HRQoL of the individuals concerned and their families.

We also found age to be related to disease-specific HRQoL. This corresponds to studies on subjective health complaints, which are reported to increase from childhood to adolescence after TBI (e.g., [80]). Our findings concerning male participants reporting better HRQoL than females is also consistent with previous reports describing younger age [59] and male gender [17] or sex [16] to be significantly associated with perceptions of higher HRQoL in healthy reference groups.

Moreover, as in previous research (e.g., [18]), our findings showed no association between moderate and severe TBI and lower HRQoL. This may be explained by the fact that in some studies TBI severity and HRQoL are reported not to be linearly correlated (e.g., [81]).

The relationship between time since injury and HRQoL was positive and significantly different from zero. Yet, the explained variance was only 2%. Therefore, further research is needed, especially regarding other influencing factors (e.g., age, TBI severity, etc.).

Our results also showed that the participants with mild to severe depression and above-average post-concussion symptoms were those who had experienced their TBI more recently. This finding is in contrast to studies reporting that persistent symptoms and negative consequences after TBI in early childhood may only be observed later in life [82]. In our study, only few symptoms were reported several years after TBI, possibly due to the fact that our participants had mainly experienced a mild TBI. Several studies emphasize that for persistent symptoms, severity could be more important than the time after the TBI event or the age of the child (e.g., [83]).

Based on the literature, we also expected that HRQoL would increase with better functional recovery (e.g., [5]) and lower levels of actual cognitive, physical, or sensory problems [59]. In line with this assumption, the level of functional disability/recovery of the children and adolescents after TBI was moderately correlated with disease-specific HRQoL. Those experiencing better recovery and a lower symptom burden experienced good HRQoL, and vice versa. This finding supports previous observations of children hospitalized with brain injuries of all severities, who even after a mild TBI reported lower generic HRQoL [18]. Furthermore, in our study, individuals with actual physical, sensory, and cognitive problems reported a significantly lower HRQoL than did individuals without these problems. This finding is reflected in several studies mentioned in a meta-analysis (e.g., [18]).

In the current study, significantly lower QOLIBRI-KID/ADO total scores were reported by participants with a higher number of post-concussion symptoms, indicating lower HRQoL compared with those mentioning fewer symptoms. The proportion of children and adolescents with post-concussion symptoms (11%) is comparable to that in previous work, with a prevalence of 14 to 29% after mild TBI (e.g., [84]). Post-concussion symptoms are most noticeable within the first year after TBI, but they can persist many years post-injury [85]. The significant relationship between post-concussion symptoms and HRQoL reported in our study illustrates the importance of assessing these early on and considering this association in therapeutic contexts, even years after TBI, also in children after mild TBI.

### 4.1. Limitations

We used a retrospective convenience sample to develop the instrument, which is an accepted scientific practice when developing PROMs for patients (e.g., [18]). As in most observational studies [18], our sample consisted predominantly of pediatric individuals after uncomplicated mild TBI. Most of them had experienced the TBI more than four years previously. This lack of variance may complicate the interpretation of the psychometric results. However, we discovered that HRQoL as measured by the QOLIBRI-KID/ADO total score, was affected by time since TBI but not by TBI severity. Therefore, as our instrument is able to detect differences in HRQoL among participants after mild TBI, it should also be able to reliably and validly measure HRQoL in patients after moderate to severe TBI.

A further limitation may be the fact that we used parent reports of the children’s and adolescents’ depression and anxiety. Differences in agreement between self-ratings and parent ratings of emotional problems, especially among older children, may mean that the validity of parent ratings as a proxy for children’s self-reports may be very limited [25]. Likewise, children and adolescents whose parents reported any problems (e.g., cognitive, physical, and sensory) rated their HRQoL lower.

Another limitation of the study is that our results on TBI-specific HRQoL may be biased by self-selection (e.g., [86]), as approximately 80–90% of families did not wish to participate. We observed that parents of children who had a severe TBI and suffered serious negative consequences refused to participate because of a potential re-traumatization through taking part in the study. On the other hand, parents of children who did not experience any symptoms after TBI did not want them to participate as they felt this was neither beneficial for the study nor for their children. This may be another reason for carrying out an external validation.

Learning more about the HRQoL of a broader range of children and adolescents after more severe TBI with cognitive impairment should also be a topic for future research, as well as the investigation of patients with different care pathways (e.g., those discharged from the emergency room vs. those admitted to a hospital ward or intensive care unit) and children and adolescents from families with risk factors (e.g., lower socioeconomic status, violence as cause of TBI, etc.).

### 4.2. Outlook

The final external validation of the QOLIBRI-KID/ADO is currently being conducted, including two reworded items that were affected by age differences (as indicated by DIF), responsiveness testing, and a replication of the psychometric analyses. Furthermore, linguistic validation of the questionnaire is ongoing in English, French, Italian, and Spanish.

As a part of our research, we are also developing a parent version of the QOLIBRI-KID/ADO for use as a surrogate when children are unable to self-report on their HRQoL. Further comparisons of the QOLIBRI-KID/ADO self-reports with parental and general population assessments are also underway to increase the clinical relevance of HRQoL evaluation after pTBI. We will also provide a self- and proxy-reported version for young children (QOLIBRI-KIDDY version for children of 6–7 years of age) for longitudinal evaluation.

## 5. Conclusions

The development of the QOLIBRI-KID/ADO questionnaire for children and adolescents after TBI in a multicenter study in the German-speaking context has resulted in the first reliable and valid pTBI-specific HRQoL instrument. The questionnaire comprises six domains, including Cognition, Self, Daily Life and Autonomy, Social Relationships, Emotions, and Physical Problems. Significant sensitive relationships were observed between the QOLIBRI-KID/ADO and generic HRQoL as well as, e.g., with functional recovery, depression, anxiety, and post-concussion symptoms, confirming the validity of the new instrument. Internal consistency of the instrument concerning these aforementioned clinical criteria as well as different symptom domains was satisfactory to excellent. Analyses also revealed significant associations between pre-injury factors (age, sex, education level of parents) and injury-related characteristics and HRQoL. The QOLIBRI-KID/ADO questionnaire will be a useful tool for assessing self-reported disease-specific HRQoL after TBI, collecting information that is not captured by currently available clinical ratings of functional recovery or PROMs in the field of pTBI. In addition to research, assessing TBI-specific HRQoL will enable health authorities to address broader areas of public health policy. It is also important for interpreting the outcomes in daily clinical routine. Furthermore, this instrument is especially relevant for clinical and rehabilitation contexts, for evaluating the effectiveness of interventions and health care programs, for tailoring them, and for understanding the unmet needs of affected individuals after TBI.

## Figures and Tables

**Table 1:** Demographic and clinical characteristics of participants.

Variable	Group	*N* (%)
Gender	Female	120 (40%)
	Male	179 (60%)
	Non-binary	1(0%)
Age	Children (8–12 years)	152 (51%)
	Adolescents (13–17 years)	148 (49%)
Parents’ Highest Level of Education	Primary school	1 (0%)
	Secondary school	57 (19%)
	Vocational school/college	39 (13%)
	University	189 (63%)
	Data missing	14 (5%)
TBI Severity	Mild	215 (72%)
	Moderate	25 (8%)
	Severe	60 (20%)
Cerebral Lesion(s) Found in Neuroimaging	No	208 (69%)
	Yes	86 (29%)
	Data missing	6 (2%)
KOSCHI Disability/Recovery Score	3a	0 (0%)
	3b	1 (0%)
	4a	8 (3%)
	4b	22 (7%)
	5a	40 (13%)
	5b	229 (76%)
Time Since TBI	<1 year	7 (2%)
	1–<2 years	44 (15%)
	2–<4 years	81 (27%)
	4–10 years	167 (56%)
	Data missing	1 (0%)
Physical, Sensory, and Cognitive Problems after TBI	No	196 (65%)
	Yes	100 (33%)
	Data missing	4 (1%)
Cognitive Problems after TBI	No	218 (73%)
	Yes	77 (26%)
	Data missing	5 (2%)
Learning Rate (RAVLT)	Below average (*M* − 1*SD*)	124 (41%)
	Average (*M*)	34 (11%)
	Above average (*M* + 1*SD*)	140 (47%)
	Data missing	2 (1%)
Anxiety (GAD-7)	No anxiety (1–4)	208 (69%)
	Mild to severe anxiety (≥5)	85 (28%)
	Data missing	7 (2%)
Depression (PHQ-9)	No depression (1–4)	187 (62%)
	Mild to severe depression (≥5)	106 (35%)
	Data missing	7 (2%)
Post-Concussion Symptoms (PCSI-SR8/-SR13)	Fewer symptoms (*M* − 1*SD*)	9 (3%)
	Average (*M*)	214 (71%)
	More symptoms (*M* + 1*SD*)	34 (11%)
	Data missing	43 (14%)

Note. *N* = absolute frequencies, % = percent, TBI = traumatic brain injury, KOSCHI = King’s Outcome Scale for Closed Head Injury, RAVLT = Rey Auditory Verbal Learning Test, GAD-7 = Generalized Anxiety Disorder 7, PHQ-9 = Patient Health Questionnaire 9, PCSI = Post-Concussion Symptom Inventory.

**Table 2:** Frequency of symptoms in individuals after mild or moderate to severe TBI.

Group	Mild TBI *N* = 215 (100%)	Moderate/Severe TBI *N* = 85 (100%)
Low Functional Recovery ^1^	11 (5%)	20 (24%)
Physical, Sensory, and Cognitive Problems ^2^	55 (26%)	45 (54%)
Mild to Severe Anxiety ^3^	53 (25%)	32 (39%)
Mild to Severe Depression ^4^	65 (31%)	41 (49%)
Post-Concussion Symptoms ^5^	24 (12%)	9 (14%)
Low Learning Rate ^6^	81 (38%)	43 (51%)

Note. *N* = absolute frequencies; % = percentage; ^1^ KOSCHI score < 5; ^2^ based on parent-report; ^3^ GAD-7 score ≥ 5; ^4^ PHQ-9 score ≥ 5; ^5^ PCSI-SR8/-SR13 symptoms (*M* + 1*SD =* impaired); ^6^ RAVLT low learning rate (*M* − 1*SD* = impaired).

**Table 3:** Psychometric properties of the 43 items before psychometric item reduction.

Scale (Cronbach’s α ^a^)	Item Origin	Item	Changes in α If Omitted ^a^	CITC ^a^	Item Loadings (CFA)
Cognition(0.70)	QOLIBRI	Concentration	−0.06	0.59	0.60
	QOLIBRI	Talking to Others	−0.03	0.48	0.67
	QOLIBRI	Remembering	−0.04	0.50	0.50
	QOLIBRI	Thinking Speed	−0.07	0.66	0.68
	QOLIBRI	Planning	−0.03	0.45	0.60
	QOLIBRI	Orientation	−0.01	0.39	0.52
	QOLIBRI	Decision Between Two Things	−0.01	0.40	0.56
Self (0.78)	QOLIBRI	Appearance	−0.08	0.77	0.74
	QOLIBRI	Self-Esteem	−0.08	0.77	0.81
	QOLIBRI	Accomplishment	0.00	0.49	0.68
	QOLIBRI	Energy	−0.01	0.52	0.70
	QOLIBRI	Future	−0.05	0.64	0.66
Daily Life and Autonomy (0.73)	QOLIBRI	Manage at School	−0.02	0.47	0.62
	New	Decision Making	−0.03	0.50	0.56
	QOLIBRI	Daily Independence	0.00	0.31	0.40
	QOLIBRI	Domestic Activities ^b^	0.01	0.29	0.37
	New	Ability to Move	−0.03	0.55	0.61
	QOLIBRI	Getting Out and About	−0.03	0.53	0.55
	New	Helped by Family ^c^	−0.02	0.53	0.65
	QOLIBRI	Social Activities	−0.04	0.57	0.68
	New	Support from Others	−0.03	0.56	0.70
	New	Finish What You Had Planned ^c^	−0.01	0.42	0.51
Social Relationships (0.77)	QOLIBRI	Family Relationship	0.00	0.35	0.45
	New	Safe and Secure ^c^	−0.02	0.54	0.64
	QOLIBRI	Relationship with Friends	−0.03	0.63	0.66
	QOLIBRI	Attitudes of Others	−0.04	0.67	0.69
	New	Demands from Others	−0.02	0.49	0.68
	New	Center of Attention ^c^	0.02	0.30	0.44
	New	Number of Friends ^d^	−0.02	0.55	0.57
	New	Friendships	−0.03	0.64	0.72
	New	Open up to Others	−0.03	0.56	0.66
	New	Internet/Social Media ^d^	−0.03	0.54	0.58
Emotions (0.70)	QOLIBRI	Anger	−0.06	0.58	0.59
	QOLIBRI	Anxiety	−0.05	0.55	0.62
	QOLIBRI	Boredom ^c^	0.01	0.37	0.42
	QOLIBRI	Sadness	−0.08	0.63	0.66
	QOLIBRI	Loneliness	−0.08	0.63	0.72
Physical Problems (0.73)	QOLIBRI	Headaches	−0.06	0.65	0.68
	New ^+^	Pain	−0.07	0.68	0.62
	QOLIBRI	TBI Effects	0.01	0.36	0.72
	QOLIBRI	Clumsiness	−0.04	0.57	0.71
	QOLIBRI	Seeing/Hearing	−0.04	0.55	0.68
	QOLIBRI	Other Injuries	−0.03	0.53	0.54

Note. CITC = corrected item-total correlations, CFA = confirmatory factor analysis. Negative values in “Changes in α if omitted” indicate a decrease in a scale’s Cronbach’s α if this item is omitted, ^a^ Cronbach’s α for scales and CITC values on the basis of the assumed scale structure and 43 items, ^b^ Items that were excluded due to low factor loading (CFA), ^c^ Items that were excluded due to increase in Cronbach’s α if omitted, ^d^ Items that were excluded to reduce the questionnaire’s length, ^+^ In the adult QOLIBRI version, Pain and Headaches are assessed in a single question.

**Table 4:** Descriptive statistics for the total and scale scores of the QOLIBRI-KID/ADO, PedsQL, PHQ-9, and GAD-7.

Instrument	Scale	*N*	*M*	*SD*	*SK*
QOLIBRI-KID/ADO	Cognition	300	75.20	13.45	−0.70
	Self	299	80.09	15.61	−0.99
	Daily Life and Autonomy	298	86.63	11.42	−1.47
	Social Relationships	300	81.70	12.75	−0.91
	Emotions	300	52.99	24.05	−0.08
	Physical Problems	300	63.54	21.66	−0.44
	Psychosocial Score	297	73.33	11.62	−0.42
	Total Score	297	74.75	11.27	−0.53
PedsQL	Physical Functioning	300	83.35	13.43	−1.09
	Psychosocial Functioning	298	77.80	13.82	−0.84
	Total Functioning	298	79.19	12.78	−0.85
GAD-7	Total Score	293	3.5	3.32	1.31
PHQ-9	Total Score	293	4.32	3.81	1.26

Note. *N* = absolute frequencies, *M* = mean, *SD* = standard deviation, *SK* = skewness. Negative values indicate left-skewed distributions. PedsQL = Pediatric Quality of Life Inventory, GAD-7 = Generalized Anxiety Disorder 7, PHQ-9 = Patient Health Questionnaire 9.

**Table 5:** Cronbach’s α of total and scale scores of the QOLIBRI-KID/ADO for individuals with different TBI severity and with and without any actual symptom burden.

		Scale						
Subgroups	*N*	Cognition	Self	Daily Life and Autonomy	Social Relationships	Emotions	Physical Problems	Total Score
Total		0.70	0.80	0.70	0.73	0.71	0.73	0.89
Moderate/Severe TBI	85	0.73	0.77	0.70	0.76	0.64	0.72	0.89
Mild TBI	215	0.67	0.78	0.68	0.71	0.74	0.74	0.88
Low Functional Recovery ^1^	31	0.57	0.80	0.62	0.66	0.76	0.57	0.84
Full Functional Recovery ^1^	269	0.71	0.78	0.69	0.74	0.71	0.74	0.89
Physical, Sensory, and Cognitive Problems ^2^	100	0.64	0.78	0.67	0.71	0.72	0.74	0.87
No Physical, Sensory, or Cognitive Problems ^2^	196	0.71	0.78	0.70	0.73	0.70	0.68	0.88
Cognitive Problems ^2^	77	0.66	0.80	0.65	0.73	0.73	0.73	0.88
No Cognitive Problems ^2^	218	0.69	0.77	0.71	0.72	0.71	0.70	0.88
Mild to Severe Anxiety ^3^	85	0.74	0.79	0.71	0.70	0.68	0.67	0.87
No Anxiety ^3^	208	0.64	0.76	0.62	0.72	0.72	0.72	0.88
Mild to Severe Depression ^4^	106	0.68	0.77	0.65	0.71	0.70	0.69	0.86
No Depression ^4^	187	0.66	0.77	0.62	0.71	0.72	0.71	0.88
Post-Concussion Symptoms ^5^	34	0.68	0.79	0.72	0.70	0.49	0.49	0.84
No Post-Concussion Symptoms ^5^	223	0.67	0.72	0.58	0.67	0.70	0.70	0.85
Low Learning Rate ^6^	124	0.73	0.81	0.71	0.75	0.73	0.73	0.90
High Learning Rate ^6^	140	0.67	0.74	0.66	0.72	0.72	0.71	0.87

Note. *N* = absolute frequencies; ^1^ KOSCHI (low = 3a/b, 4a/b, high = 5a/5b; ^2^ based on parent-report; ^3^ GAD-7 (mild to severe ≥ 5, no < 5); ^4^ PHQ-9 (mild to severe ≥ 5, no < 5); ^5^ PCSI-SR8/-SR13 (symptoms = impaired: *M* + 1*SD*, not impaired = *M* and *M* − 1*SD*); ^6^ RAVLT (low learning rate = *M* − 1*SD*, not impaired: *M* and *M* + 1*SD*).

**Table 6:** Test-retest reliability.

Scale	ICC(2,1)	Sem	MDC
Cognition	0.644 (0.570–0.708)	0.55	1.53
Self	0.513 (0.403–0.609)	0.58	1.60
Daily Life and Autonomy	0.518 (0.426–0.599)	0.46	1.27
Social Relationships	0.424 (0.315–0.523)	0.55	1.52
Emotions	0.468 (0.335–0.583)	0.88	2.44
Physical Problems	0.539 (0.441–0.624)	0.93	2.58
Total score	0.577 (0.541–0.611)	0.68	1.88

Note. ICC(2,1) = intraclass correlation coefficient, SEm = standard error of measurement, MDC = minimal detectable change.

**Table 7:** Pearson correlation coefficients between the QOLIBRI-KID/ADO total and scale scores with the PedsQL, GAD-7, and PHQ-9.

	PedsQL				GAD-7	PHQ-9
QOLIBRI-KID/ADO	Physical Functioning	Social Scale Score	Psycho- Social Functioning	Total Score		
Cognition	0.31	0.36	0.49	0.48	−0.26	−0.35
Self	0.19	0.26	0.45	0.37	−0.24	−0.29
Daily Life and Autonomy	0.41	0.54	0.54	0.55	−0.24	−0.30
Social Relationships	0.27	**0.47**	0.49	0.47	−0.23	−0.26
Emotions	0.31	0.25	0.43	0.43	−0.18	−0.15
Physical Problems	0.53	0.38	0.49	0.54	−0.21	−0.27
Psychosocial Score	0.37	0.43	**0.60**	0.59	−0.30	−0.33
Total Score	0.50	0.53	0.67	**0.67**	**−0.31**	**−0.36**

Note. Values in bold emphasize correlations considered for convergent/construct (PedsQL) and discriminant (GAD-7 and PHQ-9) validity. Negative values indicate lower TBI-specific HRQoL (lower QOLIBRI-KID/ADO scores) associated with symptoms increase. PedsQL = Pediatric Quality of Life Inventory, GAD-7 = Generalized Anxiety Disorder 7, PHQ-9 = Patient Health Questionnaire 9.

**Table 8:** Mean QOLIBRI-KID/ADO scores for groups of participants stratified by sociodemographic and clinical characteristics.

	QOLIBRI-KID/ADO					
Group	*M*	*SD*	*t*	*df*	*p*	*d*
Children	76.85	10.71	3.34	291.16	<0.001 *	0.39
Adolescents	72.55	11.46				
Female	72.83	11.85	2.52	234.98	0.006 *	0.31
Male	76.21	10.52				
TBI within the last four years	72.76	11.70	3.03	288.18	0.003 *	0.35
TBI more than four years ago	76.69	10.57				
Low Functional Recovery ^1^	86.65	1.64	2.39	37.18	0.012 *	0.45
Full Functional Recovery ^1^	73.87	11.51				
Physical, Sensory, and Cognitive problems ^2^	70.58	11.67	4.04	151.84	<0.001 *	0.54
No Physical, Sensory, and Cognitive problems ^2^	76.48	10.65				
Mild to Severe Anxiety^3^	69.93	11.34	4.26	152.79	<0.001 *	0.56
No Anxiety ^3^	75.21	11.03				
Mild to Severe Depression ^4^	68.83	11.22	5.35	211.40	<0.001 *	0.66
No Depression ^4^	76.03	10.76				
Post-Concussion Symptoms ^5^	59.85	10.10	9.40	42.64	<0.001 *	1.80
No Post-Concussion Symptoms ^5^	77.22	9.55				
Low Learning Rate ^6^	75.01	11.77	0.65	296.26	0.516	0.07
Higher Learning Rate ^6^	74.16	10.68				

Note. *M* = mean, *SD* = standard deviation, *df* = degrees of freedom, *t* = *t*-value, *p* = *p*-value (* = *p* < 0.05), *d* = Cohen’s *d*. ^1^ KOSCHI (low = 3a/b, 4a/b, high = 5a/5b; ^2^ based on parent-report; ^3^ GAD-7 (mild to severe ≥ 5, no < 5); ^4^ PHQ-9 (mild to severe ≥ 5, no < 5); ^5^ PCSI-SR8/-SR13 (symptoms = impaired: *M* + 1*SD*, not impaired = *M* and *M* − 1*SD*); ^6^ RAVLT (low learning rate = *M* − 1*SD*, not impaired: *M* and *M* + 1*SD*).

## Data Availability

The data presented in this study are available on request from the corresponding authors. Data are not publicly available for reasons of data protection.

## Supplementary Materials

The following supporting information can be downloaded at: https://www.mdpi.com/article/10.3390/jcm12154898/s1, S1—QOLIBRI-KID/ADO item pool generation and item reduction. References [87,88,89,90,91,92,93,94,95,96,97,98,99,100,101,102] are cited in the Supplementary Materials.

## Appendix A. Sample Description for Age Groups

**Table A1:** Demographic and clinical characteristics of the participants by age group.

Variable	Group	Children (*N*, %)	Adolescents (*N*, %)
Gender	Female	58 (38%)	62 (42%)
	Male	94 (62%)	85 (57%)
	Non-binary	0 (0%)	1 (1%)
Age	*M*	10.63	15.24
	*SD*	1.40	1.47
	Min	8.00	13.00
	Max	12.92	17.92
Parents’ Highest Level of Education	Primary school	0 (0%)	1 (1%)
	Secondary school	33 (22%)	24 (16%)
	Occupational school	20 (14%)	19 (12%)
	University	92 (62%)	97 (64%)
	Unknown or missing data	3 (2%)	11 (7%)
TBI Severity	Mild	106 (70%)	109 (74%)
	Moderate	16 (11%)	9 (6%)
	Severe	30 (20%)	30 (20%)
Lesion(s)	No	108 (71%)	100 (68%)
	Yes	43 (28%)	43 (29%)
	Data missing	1 (1%)	5 (3%)
KOSCHI Disability Score	3a	0 (0%)	0 (0%)
	3b	1 (1%)	0 (0%)
	4a	3 (2%)	5 (3%)
	4b	4 (3%)	18 (12%)
	5a	15 (10%)	25 (17%)
	5b	129 (85%)	100 (68%)
Time Since TBI	<1 year	4 (3%)	3 (2%)
	1–<2 years	24 (16%)	20 (14%)
	2–<4 years	45 (30%)	36 (24%)
	4–10 years	79 (52%)	88 (59%)
	Data missing	0 (0%)	1 (1%)
Physical, Sensory, and Cognitive Problems after TBI	No	97 (64%)	99 (67%)
	Yes	51 (33%)	49 (33%)
	Data missing	4 (3%)	0 (0%)
Cognitive Problems after TBI	No	108 (71%)	110 (74%)
	Yes	40 (26%)	37 (25%)
	Data missing	4 (3%)	1 (1%)
RAVLT Learning Rate	Below average	71 (47%)	53 (36%)
	Average	21 (14%)	13 (9%)
	Above average	60 (39%)	80 (54%)
	Data missing	0 (0%)	2 (1%)
GAD-7	No anxiety (1–4)	98 (64%)	110 (74%)
	Mild to severe anxiety (≥5)	49 (32%)	36 (24%)
	Data missing	5 (3%)	2 (1%)
PHQ-9	No depression (1–4)	98 (64%)	89 (60%)
	Mild to severe depression (≥5)	49 (32%)	57 (39%)
	Data missing	5 (3%)	2 (1%)
PCSI-SR8/-SR13	Fewer symptoms (below average)	0 (0%)	9 (6%)
	Average	117 (77%)	97 (65%)
	More symptoms (above average)	15 (10%)	19 (13%)
	Missing	20 (13%)	23 (16%)

Note. *N* = absolute frequencies, % = percent, *M* = mean, *SD* = standard deviation, Min = Minimum, Max = Maximum, TBI = traumatic brain injury, KOSCHI = King’s Outcome Scale for Closed Head Injury, RAVLT = Rey Auditory Verbal Learning Test, GAD-7 = Generalized Anxiety Disorder 7, PHQ-9 = Patient Health Questionnaire 9, PCSI = Post-Concussion Symptom Inventory.

## Appendix B. Results of DIF-Analyses of the QOLIBRI-KID/ADO Items

**Table A2:** Summary of LORDIF analyses.

Scale	Item	*p*-Value	McFadden’s *R*²
Cognition	Concentration (*)	0.033	
	Talking to Others (*)	0.753	
	Remembering (*)	0.150	
	Thinking Speed (*)	0.401	
	Planning (*)	0.395	
	Orientation (*)	0.001 ***	0.018
	Decision Between Two Things (*)	0.094	
Self	Appearance (*)	0.026	
	Self-Esteem (*)	0.345	
	Accomplishment (*)	0.418	
	Future (*)	0.755	
	Energy (*)	0.994	
Daily Life and Autonomy	Manage at School (*)	0.625	
	Decision Making	0.951	
	Daily Independence	0.003 **	0.041
	Getting Out and About (*)	0.247	
	Social Activities (*)	0.151	
	Support from Others	0.323	
	Ability to Move	0.254	
Social Relationships	Family Relationship (*)	0.111	
	Relationship with Friends (*)	0.130	
	Attitudes of Others (*)	0.051	
	Friendships	0.083	
	Open up to Others	0.994	
	Demands from Others	0.022	
Emotions	Anger (*)	0.298	
	Anxiety (*)	0.020	
	Sadness (*)	0.309	
	Loneliness (*)	0.955	
Physical Problems	TBI Effects (*)	0.605	
	Headaches (+)	0.857	
	Pain (+)	0.399	
	Clumsiness (*)	0.640	
	Seeing/Hearing (*)	0.012	
	Other Injuries (*)	0.451	

Note. *p*-values refer to a χ^2^-test between LORDIF models regressing on responses to an item. One regression model only used scale scores as a predictor, whereas the other applied scale score, the age category, and the age category–ability interaction. McFadden’s R^2^ is only reported for items with significant differences in the comparison of the models. ** = *p*-value < 0.01, *** = *p*-value < 0.001. (*) = Items corresponding to the items in the adult QOLIBRI version. (+) = Pain and Headaches are assessed using a single question in the adult QOLIBRI version.

## Appendix C. Item and Scale Values of the QOLIBRI-KID/ADO Questionnaire by Age Group

**Table A3:** Descriptive statistics for items of the QOLIBRI-KID/ADO.

Scale	Item	*M*	*SD*	% Missing	*SK*	% Floor	% Ceiling
Cognition	Concentration (*)	4.07	0.87	1.67	−0.89	5	78
	Talking to Others (*)	3.76	0.86	1.67	−0.57	6	64
	Remembering (*)	4.39	0.82	0	−1.47	3	87
	Thinking Speed (*)	4.73	0.57	0	−2.12	0	94
	Planning (*)	3.85	1.01	1	−0.64	8	64
	Orientation (*)	3.94	1.03	0.33	−0.85	8	69
	Decision Between Two Things (*)	3.94	0.91	0	−0.71	7	73
Self	Appearance (*)	4.20	0.90	0.33	−1.06	4	79
	Self-Esteem (*)	4.59	0.59	0	−1.32	1	96
	Accomplishment (*)	4.02	0.88	0.33	−0.68	4	74
	Future (*)	4.24	0.89	0.67	−1.16	5	82
	Energy (*)	3.45	1.00	0	−0.55	14	52
Daily Life and Autonomy	Manage at School (*)	4.16	0.83	0.33	−0.87	4	82
	Decision Making	4.31	0.79	1.33	−1.21	2	85
	Daily Independence	4.17	0.82	0.67	−0.89	2	80
	Getting Out and About (*)	4.56	0.66	0	−1.70	1	94
	Social Activities (*)	4.43	0.87	0.67	−1.83	4	87
	Support from Others	4.83	0.51	2	−3.83	1	95
	Ability to Move	4.52	0.85	1.33	−1.91	4	86
Social Relationships	Family Relationship (*)	3.95	0.80	0	−0.76	4	77
	Relationship with Friends (*)	3.96	0.96	1	−0.64	8	69
	Attitudes of Others (*)	4.65	0.71	0.67	−2.26	2	92
	Friendships	4.61	0.68	0	−2.02	2	94
	Open up to Others	4.44	0.69	0.67	−1.19	1	91
	Demands from Others	4.15	0.88	0.67	−0.97	6	81
Emotions	Anger (*)	2.94	1.33	0	0.14	42	35
	Anxiety (*)	2.97	1.20	0	0.04	35	33
	Sadness (*)	3.16	1.26	0.33	−0.06	30	38
	Loneliness (*)	4.17	1.2	0	−1.37	11	77
Physical Problems	TBI Effects (*)	3.98	1.34	2.67	−0.99	17	66
	Headaches (+)	3.12	1.23	0.33	−0.10	31	39
	Pain (+)	3.42	1.44	1.33	−0.41	29	53
	Clumsiness (*)	3.03	1.44	0	0.01	41	41
	Seeing/Hearing (*)	3.77	1.38	0.33	−0.72	22	62
	Other Injuries (*)	3.19	1.33	1.33	−0.17	30	42

Note. *M* = mean, *SD* = standard deviation, *SK* = skewness, % = percent. Negative values indicate left-skewed distributions. (*) Items corresponding to items in the adult QOLIBRI version. (+) In the adult QOLIBRI version, Pain and Headaches were assessed in a single question.

**Table A4:** QOLIBRI-KID/ADO item descriptives for the children’s (KID) version.

Scale	Item	*M*	*SD*	% Missing	*SK*	% Floor	% Ceiling
Cognition	Concentration (*)	4.24	0.82	0	−0.9	4	84
	Talking to Others (*)	3.9	0.77	0	−0.09	2	69
	Remembering (*)	4.57	0.66	0	−1.38	1	92
	Thinking Speed (*)	4.74	0.58	0	−2.27	1	94
	Planning (*)	4.02	0.97	0	−0.78	5	71
	Orientation (*)	4.26	0.84	0	−0.98	3	82
	Decision Between Two Things (*)	4.05	0.92	0	−0.92	7	78
Self	Appearance (*)	4.45	0.77	0	−1.38	1	88
	Self-Esteem (*)	4.7	0.5	0	−1.35	0	98
	Accomplishment (*)	4.32	0.76	0	−0.79	1	85
	Future (*)	4.5	0.66	0.66	−1.11	1	91
	Energy (*)	3.47	0.98	0	−0.58	12	52
Daily Life and Autonomy	Manage at School (*)	4.24	0.81	0	−0.89	4	84
	Decision Making	4.39	0.74	0	−1.26	1	89
	Daily Independence	4.23	0.82	0	−0.87	2	82
	Getting out and About (*)	4.6	0.66	0	−1.64	1	93
	Social Activities (*)	4.58	0.78	0	−2.5	3	93
	Support from Others	4.79	0.53	1.97	−2.66	1	94
	Ability to Move	4.59	0.8	0.66	−2.14	4	89
Social Relationships	Family Relationship (*)	4.05	0.77	0	−0.69	3	80
	Relationship with Friends (*)	4.29	0.83	0	−1.06	3	84
	Attitudes of Others (*)	4.71	0.63	0.66	−2.42	2	94
	Friendships	4.72	0.58	0	−2.36	1	96
	Open up to Others	4.55	0.68	0	−1.8	1	94
	Demands from Others	4.34	0.73	0	−1.22	2	91
Emotions	Anger (*)	2.85	1.35	0	0.19	43	32
	Anxiety (*)	2.88	1.22	0	0.05	37	30
	Sadness (*)	3.27	1.27	0	−0.14	26	40
	Loneliness (*)	4.08	1.25	0	−1.17	12	71
Physical Problems	TBI Effects (*)	3.86	1.42	1.97	−0.85	20	63
	Headaches (+)	3.11	1.26	0.66	−0.13	31	39
	Pain (+)	3.43	1.49	1.32	−0.45	30	55
	Clumsiness (*)	2.93	1.5	0	0.11	45	39
	Seeing/Hearing (*)	3.85	1.38	0	−0.81	20	64
	Other Injuries (*)	3.02	1.39	1.32	0.04	38	36

Note. *M* = mean, *SD* = standard deviation, *SK* = skewness, % = percent. (*) = Items corresponding to items in the adult QOLIBRI version. (+) = Pain and Headaches are assessed using a single question in the adult QOLIBRI version.

**Table A5:** QOLIBRI-KID/ADO item descriptives for the adolescents’ (ADO) version.

Scale	Item	*M*	*SD*	% Missing	*SK*	% Floor	% Ceiling
Cognition	Concentration (*)	3.9	0.89	3.38	−0.88	6	72
	Talking to Others (*)	3.62	0.92	3.38	−0.74	9	59
	Remembering (*)	4.2	0.92	0	−1.27	5	82
	Thinking Speed (*)	4.73	0.55	0	−1.92	0	95
	Planning (*)	3.66	1.03	2.03	−0.51	11	57
	Orientation (*)	3.61	1.1	0.68	−0.59	13	56
	Decision Between Two Things (*)	3.83	0.89	0	−0.53	8	69
Self	Appearance (*)	3.95	0.96	0.68	−0.78	7	71
	Self-Esteem (*)	4.47	0.65	0	−1.13	1	94
	Accomplishment (*)	3.71	0.9	0.68	−0.54	7	62
	Future (*)	3.98	1	0.68	−0.85	9	72
	Energy (*)	3.43	1.02	0	−0.51	16	51
Daily Life and Autonomy	Manage at School (*)	4.09	0.84	0.68	−0.85	5	80
	Decision Making	4.22	0.84	2.70	−1.13	3	81
	Daily Independence	4.1	0.83	1.35	−0.91	3	79
	Getting out and About (*)	4.53	0.66	0	−1.76	1	95
	Social Activities (*)	4.27	0.94	1.35	−1.36	5	81
	Support from Others	4.88	0.5	2.03	−5.26	1	96
	Ability to Move	4.46	0.9	2.03	−1.7	4	83
Social Relationships	Family Relationship (*)	3.86	0.83	0	−0.78	6	74
	Relationship with Friends (*)	3.61	0.98	2.03	−0.27	13	55
	Attitudes of Others (*)	4.59	0.77	0.68	−2.06	3	89
	Friendships	4.49	0.76	0	−1.71	3	91
	Open up to Others	4.33	0.7	1.35	−0.66	1	87
	Demands from Others	3.95	0.98	1.35	−0.65	9	70
Emotions	Anger (*)	3.04	1.3	0	0.09	40	38
	Anxiety (*)	3.05	1.18	0	0.05	34	35
	Sadness (*)	3.04	1.25	0.68	0.03	35	36
	Loneliness (*)	4.26	1.14	0	−1.59	11	82
Physical Problems	TBI Effects (*)	4.1	1.25	3.38	−1.12	14	69
	Headaches (+)	3.12	1.21	0	−0.07	30	38
	Pain (+)	3.41	1.39	1.35	−0.35	28	51
	Clumsiness (*)	3.14	1.38	0	−0.07	36	43
	Seeing/Hearing (*)	3.69	1.38	0.68	−0.63	24	61
	Other Injuries (*)	3.36	1.24	1.35	−0.36	23	48

Note. *M* = mean, *SD* = standard deviation, *SK* = skewness, % = percent. (*) = Items that correspond to items in the adult QOLIBRI version. (+) = Pain and Headaches are assessed using a single question in the adult QOLIBRI version.

**Table A6:** Descriptive statistics for QOLIBRI-KID/ADO, PedsQL, PHQ-9, and GAD-7 questionnaires by age group.

		Children				Adolescents			
Instrument	Scale	*N*	*M*	*SD*	*SK*	*N*	*M*	*SD*	*SK*
QOLIBRI-KID/ADO	Cognition	152	78.64	11.64	−0.45	148	71.67	14.29	−0.69
	Self	152	86.35	11.83	−0.93	147	73.62	16.41	−0.80
	Daily Life and Autonomy	152	88.71	10.2	−1.35	146	84.47	12.24	−1.46
	Social Relationships	152	84.05	12.24	−1.01	148	79.28	12.85	−0.85
	Emotions	152	52.56	25.02	−0.22	148	53.43	23.08	0.10
	Physical Problems	152	61.94	23.47	−0.32	148	65.18	19.58	−0.54
	Psychosocial score	152	75.37	11.1	−0.33	145	71.19	11.8	−0.47
	Total score	152	76.85	10.71	−0.43	145	72.55	11.46	−0.59
PedsQL	Physical functioning	147	82.60	13.82	−1.03	146	84.12	13.02	−1.14
	Psychosocial functioning	152	79.10	13.67	−1.05	148	76.44	13.88	−0.65
	Total functioning	147	79.98	12.91	−1.03	146	78.37	12.64	−0.66
PHQ-9	Total score	147	3.94	3.35	1.09	146	4.70	4.19	1.23
GAD-7	Total score	147	3.56	3.07	0.88	146	3.45	3.56	1.57

Note. *N* = absolute frequencies, *M* = mean, *SD* = standard deviation, *SK* = skewness, PedsQL = Pediatric Quality of Life Inventory, GAD-7 = Generalized Anxiety Disorder 7, PHQ-9 = Patient Health Questionnaire 9.

## Appendix D. Internal Consistencies on Item Level

**Table A7:** Internal consistency of the QOLIBRI-KID/ADO scales on item level.

Dimension	Item	α If Item Omitted	CITC
Cognition	Concentration (*)	0.65	0.60
	Talking to Others (*)	0.68	0.48
	Remembering (*)	0.67	0.50
	Thinking Speed (*)	0.63	0.66
	Planning (*)	0.68	0.45
	Orientation (*)	0.69	0.39
	Decision Between Two Things (*)	0.69	0.40
Self	Appearance (*)	0.70	0.77
	Self-Esteem (*)	0.70	0.77
	Accomplishment (*)	0.78	0.48
	Future (*)	0.74	0.64
	Energy (*)	0.77	0.52
Daily Life and Autonomy	Manage at School (*)	0.68	0.40
	Decision Making	0.65	0.51
	Daily Independence	0.70	0.28
	Getting out and About (*)	0.65	0.56
	Social Activities (*)	0.63	0.63
	Support from Others	0.67	0.46
	Ability to Move	0.64	0.61
Social Relationships	Family Relationship (*)	0.74	0.37
	Relationship with Friends (*)	0.68	0.63
	Attitudes of Others (*)	0.66	0.69
	Friendships	0.68	0.61
	Open up to Others	0.69	0.55
	Demands from Others	0.72	0.48
Emotions	Anger (*)	0.69	0.67
	Anxiety (*)	0.68	0.67
	Sadness (*)	0.62	0.63
	Loneliness (*)	0.65	0.64
Physical Problems	TBI Effects (*)	0.73	0.74
	Headaches (+)	0.68	0.67
	Pain (+)	0.67	0.66
	Clumsiness (*)	0.69	0.69
	Seeing/Hearing (*)	0.69	0.69
	Other Injuries (*)	0.70	0.70

Note. α = Cronbach’s Alpha, CITC = corrected item-total correlation for items and their corresponding scale. (*) = Items that correspond to items in the adult QOLIBRI version. (+) = Pain and Headaches are assessed using a single question in the adult QOLIBRI version.

## Appendix E. Model Parameters of the CFA on the QOLIBRI-KID/ADO Factors

**Table A8:** Fit indices and model comparison of the one-level and the two-level factorial solution.

								Model Comparison			
No. of Levels	*χ*²	*p*	CFI	TLI	RMSEA (CI90%)	SRMR	*df*	*χ*²	Δ*χ*²	Δ*df*	*P*
One	874.64	<0.001 ***	0.97	0.97	0.05 (0.04–0.05)	0.08	545	874.64	-	-	-
Two	1117.21	<0.001 ***	0.95	0.95	0.06 (0.06–0.07)	0.09	554	1117.21	116.97	9	<0.001 ***

Note. χ^2^-test of significance employs scaled statistics, whereas other statistics are unscaled. CFI = comparative fit index, TLI = Tucker–Lewis index, RMSEA (CI_90%_) = root mean square error of approximation with 90% confidence interval, SRMR = Standardized Root Mean Square Residual, *df* = degrees of freedom*, χ*^2^ = chi-square statistics, Δχ^2^ = difference in chi-square statistics after Satorra–Bentler (2001) correction, Δdf = difference in degrees of freedom, *p* = *p*-value (*** = *p* < 0.001).

## Appendix F. Association of QOLIBRI-KID/ADO Questionnaire with Clinical Characteristics

**Table A9:** Mean QOLIBRI-KID/ADO total and scale score differences between participants after mild TBI and moderate to severe TBI.

Scale	*M*_mild_	*SD*_mild_	*M*_moderate-severe_	*SD*_moderate-severe_	*df*	*t*	*p*	Cohen’s *d*
Cognition	75.95	12.47	73.33	15.58	128.69	1.38	0.253	0.20
Self	79.76	15.38	80.93	16.22	147.35	−0.57	0.500	−0.07
Daily Life and Autonomy	86.99	10.78	85.75	12.92	133.06	0.78	0.439	0.11
Social Relationships	81.85	11.98	81.31	14.58	131.19	0.30	0.500	0.04
Emotions	52.14	24.68	55.15	22.37	168.91	−1.02	0.500	−0.12
Physical Problems	64.49	21.42	61.13	22.20	149.28	1.19	0.293	0.16
Total	73.49	11.35	72.93	12.33	144.12	0.36	0.180 ^a^	0.05

Note. *M* = mean, *SD* = standard deviation, *df* = degrees of freedom, *t* = *t*-value, *p* = *p*-value. All *p*-values refer to one-tailed testing. ^a^ Non-adjusted *p*-values; all other *p*-values were adjusted using the Holm–Bonferroni method.

## Appendix G. Association of Time since Injury with Neuro-Psychiatric Symptoms

**Table A10:** Mean time in years since TBI of participants with and without neuropsychiatric symptoms and statistical significance (*t*-test) of differences between groups.

Symptoms	Symptoms Present		Symptoms Absent		*t*-Test Parameters			
	*M* (*SD*)	*n*	*M* (*SD*)	*n*	*t*	*df*	*p*	*d*
Cognitive Problems after TBI	4.21 (2.69)	77	4.79 (2.89)	218	1.60	142.54	0.112	0.21
Mild to Severe Anxiety	4.21 (2.65)	85	4.86 (2.90)	208	1.85	167.03	0.066	0.23
Mild to Severe Depression	4.22 (2.83)	106	4.93 (2.83)	187	2.06	215.38	0.040 *	0.25
Post-concussion Symptoms	3.76 (2.59)	34	4.90 (2.82)	223	2.35	44.02	0.023 *	0.41

Note. Post-concussion symptoms refer to a PCSI score that is one *SD* above the respective age average (see “Instrument” section). *M* = mean time since TBI in years, *SD* = standard deviation, *df* = degrees of freedom, *t* = *t*-value, *p* = *p*-value (* = *p* < 0.05), *d* = Cohen’s *d*.
