# Supplementary material for: Quality of Life after Brain Injury in Children and Adolescents (QOLIBRI-KID/ADO)—The First Disease-Specific Self-Report Questionnaire after Traumatic Brain Injury
Source: J Clin Med. 2023 Jul 26;12(15):4898. doi: 10.3390/jcm12154898 (PMC10419542; doi:10.3390/jcm12154898)
Supplement: Supplementary file 1 [file jcm-12-04898-s001.zip › jcm-2500422-supplementary.pdf]

Quality of Life after Brain Injury in Children and Adolescents (QOLIBRI-KID/ADO)—The First Disease-Specific Self-Report Measure after Traumatic Brain Injury

Online Supplement S1— QOLIBRI-KID/ADO item pool generation and item reduction

In the first step, an item pool was compiled based on results obtained from focus group interviews with children and adolescents after TBI. For details, see Krenz et al. (2021) [1]. The item number was reduced based on relevance of content, understandability, and appropriateness of item wording. Wording and content were made comparable for the two separate age groups (8–12 years and 13–17 years).
In a next step, five national and international experts (items were translated into English following a linguistic validation procedure [2]) rated the items with respect to the above criteria, resulting in adding and exclusion of items.

Additional items were added to the item pool following a literature search for instruments related to QoL, HRQoL, and/or TBI. Five national panels of experts (Delphi panels) then determined whether the new items included an additional aspect of HRQoL after TBI and what items ultimately to keep and which to add. In Delphi panels [3], items were rated by the experts according to importance, understandability, relevance of content, appropriateness of item wording, and applicability per age group. Items with an average agreement of at least 66%—weighted with respect to the number of participants per Delphi panel—were retained.

Finally, eight children and adolescents without TBI and six after TBI participated in a cognitive debriefing procedure [2,4]. All instructions, items, and response options were investigated across the age groups. The results showed that the current wording of the items and the response categories were understandable and appropriate for each age group. Smiley symbols clarified the response categories in the younger group.

Based on this procedure, items were again revised, and questionnaire versions with reduced item numbers were established for administration in the study reported here. These procedures resulted in an 83-item and an 87-item version, which were answered by the children and adolescents, respectively. Before psychometric reduction of items, they were analyzed in terms of their theoretical congruence with the assumed structure of the scale as derived from the QOLIBRI adult version. Items had to be present in both age groups and had to be associated with following dimensions according to expert rating and the underlying measurement model of the adult QOLIBRI version [4,5]: "Cognition", "Self", "Daily Life and Autonomy", and "Social Relationships". These should be rated using satisfaction judgments, whereas items for "Emotions" and "Physical Problems" should be evaluated in terms of feeling bothered. Forty-three items matched these criteria and were thus included in the psychometric analyses. For more details, see Figure S1.

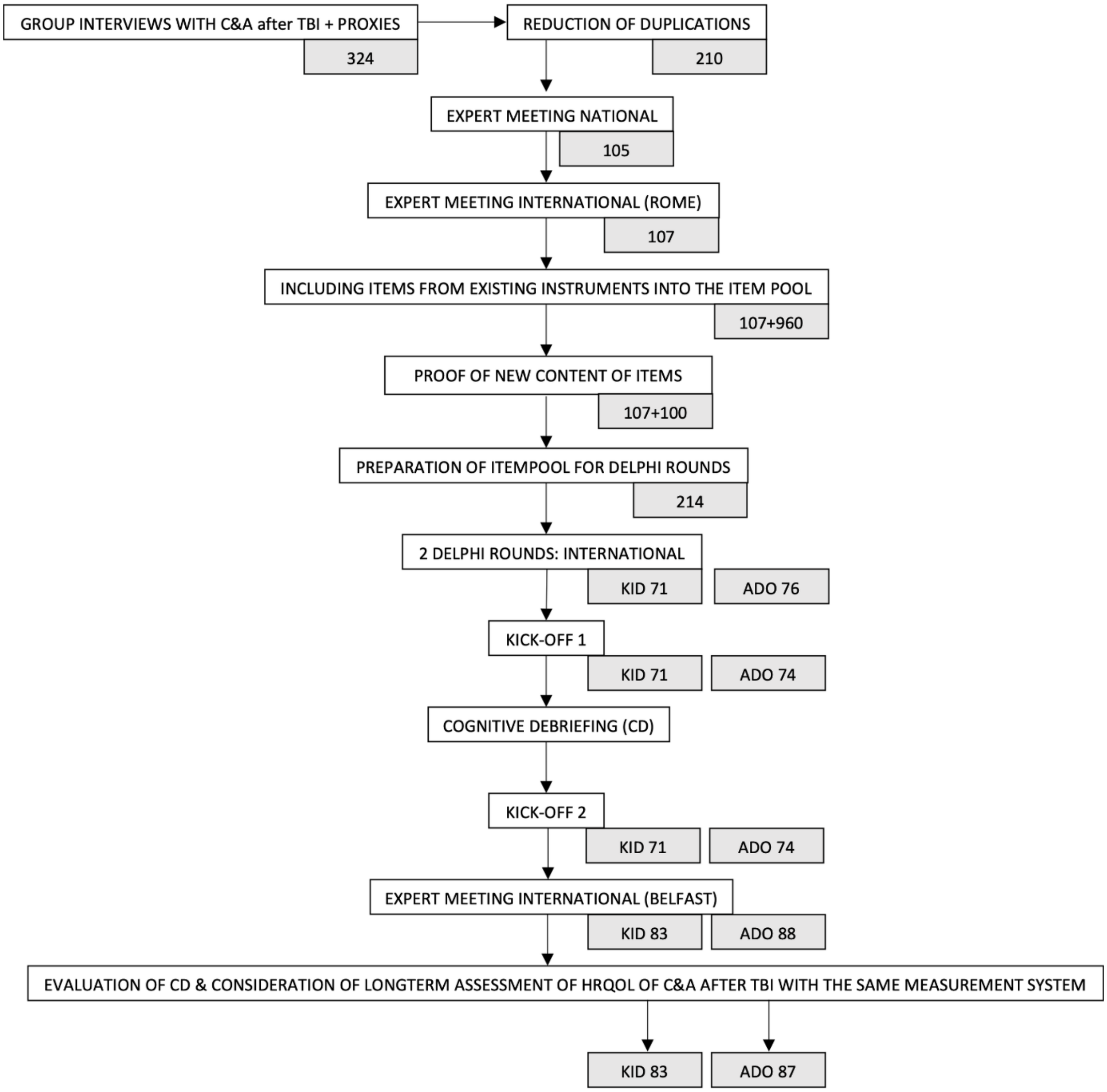

Figure S1. Process of item generation

Note: C&A = children and adolescents; TBI = traumatic brain injury.
* See acknowledgments for the names of the contributing experts.
[1] Adaptation of items from generic HRQoL instruments and other health-related instruments:
Caregivers Quality of Life Index—Cancer (CQOLC) [6]; Cerebral Palsy Quality of Life (CP QOL) [7]; Child Health Questionnaire (CHQ) [8]; Children's Health Rating Scale Items (CHRS) [9]; Health Utility Index (HUI) Mark 3 [10]; DISABKIDS [11]; Generic Children's Quality of Life Measure (GCQ) [12]; KIDSCREEN-27 [13]; KINDL [14]; PedsQL [15]; PROMIS Neuro-QOL Item Bank [16]; TNO/AZL Adult Quality Of Life (TAAQOL) [17]; Quality of Life in Epilepsy Inventory for Adolescents (QOLIE-AD-48) [18]; Quality of Life Questionnaire (17D) [19]; QOLIBRI [4,5]; Vecu et Sante Percue de l'Adolescent (VSP-A) [20].

References

1. Krenz, U.; Timmermann, D.; Gorbunova, A.; Lendt, M.; Schmidt, S.; von Steinbuechel, N. Health-Related Quality of Life after Pediatric Traumatic Brain Injury: A Qualitative Comparison between Children's and Parents' Perspectives. *PLOS ONE* **2021**, *16*, e0246514, doi:10.1371/journal.pone.0246514.
2. von Steinbuechel, N.; Rauen, K.; Krenz, U.; Wu, Y.-J.; Covic, A.; Plass, A.M.; Cunitz, K.; Mueller, I.; Bockhop, F.; Polinder, S.; et al. Translation and Linguistic Validation of Outcome Instruments for Traumatic Brain Injury Research and Clinical Practice: A Step-by-Step Approach within the Observational CENTER-TBI Study. *Journal of Clinical Medicine* **2021**.
3. Hsu, C.-C.; Sandford, B.A. He Delphi Technique: Making Sense of Consensus. *Pract. Assess. Res. Eval.* **2007**, *12*, 1–8, doi:10.7275/PDZ9-TH90.
4. von Steinbuechel, N.; Wilson, L.; Gibbons, H.; Hawthorne, G.; Höfer, S.; Schmidt, S.; Bullinger, M.; Maas, A.; Neugebauer, E.; Powell, J.; et al. Quality of Life after Brain Injury (QOLIBRI): Scale Validity and Correlates of Quality of Life. *J. Neurotrauma* **2010**, *27*, 1157–1165, doi:10.1089/neu.2009.1077.
5. von Steinbuechel, N.; Wilson, L.; Gibbons, H.; Hawthorne, G.; Höfer, S.; Schmidt, S.; Bullinger, M.; Maas, A.; Neugebauer, E.; Powell, J.; et al. Quality of Life after Brain Injury (QOLIBRI): Scale Development and Metric Properties. *J. Neurotrauma* **2010**, *27*, 1167–1185, doi:10.1089/neu.2009.1076.
6. Weitzner, M.A.; Jacobsen, P.B.; Wagner, H.Jr.; Friedland, J.; Cox, C. The Caregiver Quality of Life Index-Cancer (CQOLC) Scale: Development and Validation of an Instrument to Measure Quality of Life of the Family Caregiver of Patients with Cancer. *Qual. Life Res.* **1998**, *8*, 55–63, doi:10.1023/a:1026407010614.
7. Waters, E.; Maher, E.; Salmon, L.; Reddihough, D.; Boyd, R. Development of a Condition-Specific Measure of Quality of Life for Children with Cerebral Palsy: Empirical Thematic Data Reported by Parents and Children. *Child Care Health Dev.* **2005**, *31*, 127–135, doi:10.1111/j.1365-2214.2004.00476.x.
8. Landgraf, J.M. Child Health Questionnaire (CHQ). *Michalos AC Eds Springer Dordr.* **2014**, *Encyclopedia of Quality of Life and Well-Being Research*, doi:https://doi.org/10.1007/978-94-007-0753-5_324.
9. Maylath, N.S. Development of the Children's Health Ratings Scale. *Health Educ. Q.* **1990**, *17*, 89–97.
10. Horsman, J.; Furlong, W.; Feeny, D.; Torrance, G. The Health Utilities Index (HUI®): Concepts, Measurement Properties and Applications. *Health Qual. Life Outcomes* **2003**, *1*, 54, doi:10.1186/1477-7525-1-54.
11. Bullinger, M.; Schmidt, S.; Petersen, C.; GROUP, D.; others Assessing Quality of Life of Children with Chronic Health Conditions and Disabilities: A European Approach. *Int. J. Rehabil. Res.* **2002**, *25*, 197–206.
12. Collier, J.; MacKinlay, D.; Phillips, D. Norm Values for the Generic Children's Quality of Life Measure (GCQ) from a Large School-Based Sample. *Qual. Life Res.* **2000**, *9*, 617–623, doi:10.1023/a:1008915700210.
13. Ravens-Sieberer, U.; Auquier, P.; Erhart, M.; Gosch, A.; Rajmil, L.; Bruil, J.; Power, M.; Duer, W.; Cloetta, B.; Czemy, L.; et al. The KIDSCREEN-27 Quality of Life Measure for Children and Adolescents: Psychometric Results from a Cross-Cultural Survey in 13 European Countries. *Qual. Life Res. Int. J. Qual. Life Asp. Treat. Care Rehabil.* **2007**, *16*, 1347–1356, doi:10.1007/s11136-007-9240-2.
14. Ravens-Sieberer, U.; Bullinger, M. Assessing Health-Related Quality of Life in Chronically Ill Children with the German KINDL: First Psychometric and Content Analytical Results. *Qual. Life Res.* **1998**, *7*, 399–407, doi:10.1023/A:1008853819715.
15. Varni, J.W.; Seid, M.; Rode, C.A. The PedsQL™: Measurement Model for the Pediatric Quality of Life Inventory: *Med. Care* **1999**, *37*, 126–139, doi:10.1097/00005650-199902000-00003.

16. Cella, D.; Lai, J.-S.; Nowinski, C.J.; Victorson, D.; Peterman, A.; Miller, D.; Bethoux, F.; Heinemann, A.; Rubin, S.; Cavazos, J.E.; et al. Neuro-QOL: Brief Measures of Health-Related Quality of Life for Clinical Research in Neurology. *Neurology* **2012**, *78*, 1860–1867, doi:10.1212/WNL.0b013e318258f744.
17. Bruil, J.; Fekkes, M.; Vogels, T. The Validity and Reliability of the TAAQOL: A Health-Related Quality of Life Instruments Comprising Health Status Weighted by the Impact of Problems on Well Being. *Qual. Life Res.* **2001**, *10*, 257.
18. Cramer, J.A.; Westbrook, L.E.; Devinsky, O.; Perrine, K.; Glassman, M.B.; Camfield, C. Development of the Quality of Life in Epilepsy Inventory for Adolescents: The QOLIE-AD-48. *Epilepsia* **1999**, *40*, 1114–1121, doi:10.1111/j.1528-1157.1999.tb00828.x.
19. Apajasalo, M.; Rautonen, J.; Holmberg, C.; Sinkkonen, J.; Aalberg, V.; Pihko, H.; Siimes, M.A.; Kaitila, I.; Maekelae, A.; Erkkilae, K.; et al. Quality of Life in Pre-Adolescence: A 17-Dimensional Health-Related Measure (17D). *Qual. Life Res.* **1996**, *5*, 532–538, doi:10.1007/BF00439227.
20. Sapin, C.; Simeoni, M.-C.; El Khammar, M.; Antoniotti, S.; Auquier, P. Reliability and Validity of the VSP-A, a Health-Related Quality of Life Instrument for Ill and Healthy Adolescents. *J. Adolesc. Health* **2005**, *36*, 327–336, doi:10.1016/j.jadohealth.2004.01.016.
